# Subclinical acute kidney injury is associated with adverse outcomes in critically ill neonates and children

**DOI:** 10.1186/s13054-018-2193-8

**Published:** 2018-10-10

**Authors:** Fang Fang, Xiaohan Hu, Xiaomei Dai, Sanfeng Wang, Zhenjiang Bai, Jiao Chen, Jian Pan, Xiaozhong Li, Jian Wang, Yanhong Li

**Affiliations:** 1 Institute of Pediatric Research, Children’s Hospital of Soochow University, Suzhou, JiangSu province China; 2 Department of Nephrology, Children’s Hospital of Soochow University, Suzhou, JiangSu province China; 3 Pediatric Intensive Care Unit, Children’s Hospital of Soochow University, Suzhou, JiangSu province China

**Keywords:** Acute kidney injury, Adverse outcomes, Critically ill neonates, Critically ill children, Length of ICU stay, Mortality, Subclinical acute kidney injury, Urinary cystatin C

## Abstract

**Background:**

Research on acute kidney injury (AKI) has focused on identifying early biomarkers. However, whether AKI could be diagnosed in the absence of the classic signs of clinical AKI and whether the condition of subclinical AKI, identified by damage or functional biomarkers in the absence of oliguria or increased serum creatinine (sCr) levels, is clinically significant remains to be elucidated in critically ill children. The aims of the study were to investigate the associations between urinary cystatin C (uCysC) levels and AKI and mortality and to determine whether uCysC-positive subclinical AKI is associated with adverse outcomes in critically ill neonates and children.

**Methods:**

In this prospective cohort study, uCysC levels were serially measured during the first week after intensive care unit (ICU) admission in a heterogeneous group of patients (*n* = 510) presenting to a tertiary neonatal and pediatric ICU. The diagnosis of neonatal AKI that developed during the first week after admission was based on neonatal KDIGO criteria or sCr >1.5 mg/dL, and pediatric AKI was based on Kidney Disease: Improving Global Outcomes (KDIGO) criteria. The term “uCysC(−)” or “uCysC(+)”, indicating the absence or presence of tubular injury, was defined by the optimal peak uCysC cutoff value for predicting ICU mortality.

**Results:**

The initial and peak uCysC levels were significantly associated with AKI and mortality, and had an area under the receiver operating characteristic curve of 0.76 and 0.81, respectively, for predicting mortality. At the optimal cutoff value of 1260 ng/mg uCr, the peak uCysC displayed sensitivity of 79.2% and specificity of 72.3% for predicting mortality. Among all patients, 130 (25.5%) developed uCysC(+)/AKI(−) status during the first week after admission. The adjusted odds ratio for patients with uCysC(+)/AKI(−) status in association with an increased risk of mortality compared with that for patients with uCysC(−)/AKI(−) was 9.34 (*P* < 0.001). Patients with uCysC(+)/AKI(−) spent 2.8 times as long in the ICU as those with uCysC(−)/AKI(−) (*P* < 0.001).

**Conclusions:**

Both initial and peak uCysC levels are associated with AKI and mortality and are independently predictive of mortality in critically ill neonates and children. Subclinical AKI may occur without detectable loss of kidney function, and uCysC-positive subclinical AKI is associated with worse clinical outcomes in this population.

**Electronic supplementary material:**

The online version of this article (10.1186/s13054-018-2193-8) contains supplementary material, which is available to authorized users.

## Background

Acute kidney injury (AKI) is associated with adverse outcomes in critically ill children, such as longer hospital stay and increased mortality, [1–3]. Research on AKI has focused on identifying early biomarkers capable of detecting kidney injury before the rise in serum creatinine (sCr) being observed in recent decades, which could optimize and improve patient outcomes [4–8]. Previous studies have demonstrated that the diagnosis of clinical AKI is delayed by at least 24-48 hours compared to diagnosis of the condition identified by using novel renal biomarkers of tubular injury, such as neutrophil gelatinase-associated lipocalin (NGAL) [9, 10] and cystatin C (CysC) [10, 11]. Therefore, questions arise regarding whether AKI could be diagnosed in the absence of the classic signs of clinical AKI and whether the condition of subclinical AKI, identified by damage or functional biomarkers in the absence of oliguria or increased sCr levels, is clinically significant. Recent studies suggest that subclinical AKI, which precedes clinical AKI and is defined as tubular injury in the absence of oliguria or increased sCr levels, is still AKI [12–15] and has an increased risk of adverse outcomes in adult patients [15]. However, whether subclinical AKI may occur frequently in critically ill neonates and children and whether the development of subclinical AKI in this population is associated with negative clinical outcomes remains to be elucidated.

Cystatin C, a 13-kDa protein, is normally filtered freely and is completely reabsorbed and catabolized in the proximal tubule and therefore is not normally found in urine in significant amounts. Increased levels of CysC in urine may reflect renal tubular injury and impairment [16, 17]. Urinary CysC (uCysC) has been shown to be diagnostic of AKI in patients in the intensive care unit (ICU) [11, 18, 19]. Our previous studies demonstrated that uCysC is associated with AKI and is independently predictive of AKI in critically ill neonates [20, 21]. Increases in uCysC levels predict AKI 24–48 hours before the occurrence of diagnostic sCr increases in response to renal injury [10, 11], suggesting that uCysC may identify subclinical kidney injury, in which kidney damage occurs without the development of decreased kidney function. However, whether uCysC could provide a method of detecting subclinical AKI in patients with apparently normal sCr is unknown. It also remains unclear whether uCysC is associated with increased mortality in critically ill neonates and children with and without AKI. The aims of the study were to investigate the association between uCysC level and AKI and mortality in critically ill neonates and children, to evaluate whether uCysC could detect subclinical AKI in patients with apparently normal sCr, and to determine whether uCysC-positive subclinical AKI was associated with adverse outcomes in this population.

## Methods

### Subject selection

All patients admitted to the neonatal and pediatric intensive care unit (NICU and PICU, respectively) from July to October 2016 were eligible for this prospective study. A priori exclusion criteria were known congenital abnormality of the kidney and failure to collect urine samples before discharge from the ICU or death. This study was approved by the Institutional Review Board at the Children’s Hospital of Soochow University and performed in accordance with the Declaration of Helsinki. Informed parental consent was obtained at enrollment.

### Clinical data collection

We reviewed the medical records of eligible patients. Clinical status, medication, and therapies were recorded daily until ICU discharge or death. Sepsis, multiple organ dysfunction syndrome (MODS), shock, and disseminated intravascular coagulation (DIC) that developed during the ICU stay were diagnosed by the treating physicians, according to the criteria described previously [22, 23].

### Assessment of illness severity

The score for neonatal acute physiology (SNAP) and the score of the pediatric risk of mortality III (PRISM III), which were calculated on the day of NICU or PICU admission according to methods described in the original study [24, 25] and in our previous studies [22, 26], were used as a measure to assess illness severity.

### Diagnosis of AKI

The diagnosis of neonatal AKI that developed during the first week after admission was based on (1) the neonatal AKI Kidney Disease: Improving Global Outcome (KDIGO) classification, i.e., sCr rise ≥ 0.3 mg/dL (26.5 μmol/L) within 48 h, sCr rise ≥ 1.5 times the reference sCr within 7 days, or urine output ≤ 0 .5 mL/kg/h for 6–12 h (the reference sCr was defined as the lowest previous sCr value) [27] or (2) sCr > 1.5 mg/dL (132.6 μmol/L) sustained at least 48 h [20]. The KDIGO criteria for sCr level and urine output were applied to define pediatric AKI that developed during the first week after PICU admission [28]. Baseline sCr was defined as the lowest level obtained within 3 months prior to ICU admission [1]. When the baseline sCr measurement was unavailable, the sCr value at the time of enrollment was used. For patients with elevated sCr ≥ 1.2 mg/dL (106.1 μmol/L) at PICU admission, the lowest sCr value within 2 weeks while in the PICU was considered the baseline sCr, which was adapted from a previous study [29] and was in accordance with our previous study [22]. When the two criteria resulted in different KDIGO stages, the higher stage was chosen. Severity of AKI was characterized by KDIGO staging. Severe AKI was defined as KDIGO stage 2 or 3 [1].

### Outcomes

The primary outcome was ICU mortality defined as all-cause mortality occurring during the ICU stay, including death resulting from withdrawal of therapy. Secondary outcomes were the duration of mechanical ventilation (MV) and the ICU length of stay.

### Measurement of urinary CysC

Urine samples were collected every 48–72 hours during the first week after the first collection on the first day of being admitted to the ICU and immediately frozen and stored at -80°C. For the measurement of CysC, the urine sample was centrifuged at 1500 g at 4 °C for 10 min, and the supernatants were aliquoted and measured using an automatic biochemical analyzer (Hitachi 7600, Tokyo, Japan) and a latex enhanced immunoturbidimetry assay, as previously described [20, 21]. The detection limit for CysC was 10 ng/mL. The intra-assay and inter-assay coefficients of variation were ≤ 10%, corresponding to that reported by the manufacturer. Urinary CysC value was expressed in nanograms per milligram of urinary Cr (ng/mg uCr). The uCr level from the same aliquoted sample was measured automatically using the sarcosine oxidase method.

### Statistical analysis

Statistical analyses were performed using SPSS statistics. We first checked the assumptions of normality and homogeneity of variance. Skewed distributions are described by the median and interquartile range (IQR) and were compared using the Mann-Whitney U test or the Kruskal-Wallis H(K) test. Categorical variables are presented as counts and percentages, and Fisher’s exact test or the chi-square test was applied for comparison, as appropriate. Stepwise multivariate linear regression analyses were performed to investigate factors potentially associated with the levels of uCysC. Univariate and multivariate logistic regression analyses were performed to calculate the odds ratio (OR) and adjusted OR (AOR) with a 95% confidence interval (CI) to assess the association between variables and AKI and mortality. To analyze the predictive power, the receiver operating characteristic (ROC) curves were generated, the area under the ROC curve (AUC) was determined, and the nonparametric method of Delong was used to compare differences between AUCs. The sensitivity, specificity, positive likelihood ratio (LR+), and negative likelihood ratio (LR-) were calculated using Sigma Plot 10.0 software. Univariate and multivariate linear regression analyses were performed to investigate the association between uCysC and the duration of MV and the length of ICU stay. Continuous variables with skewed distributions were log-transformed for linear regression analysis. The two-sided alpha level was set at 0.05.

## Results

### Patient characteristics

The prospective study involved 510 patients, including 239 neonates from the NICU and 271 children between 1 month and 16 years of age from the PICU. Of the total of 517 patients admitted to the NICU and PICU during the study period, 7 were excluded because of a failure in collecting urine samples before discharge from the ICU or death. The leading cause of ICU admission in the cohort was respiratory disease (36.5%), followed by neurologic disease (11.8%). Fifty-six patients (11.0%) were clinically diagnosed with sepsis.

Of the 510 patients, 79 (15.5%) developed AKI during the first week after admission, including 43 (8.4%) who fulfilled the criteria for KDIGO stage 1, which is defined as mild AKI, and 36 (7.1%) patients who fulfilled the criteria for KDIGO stages 2 and 3, which are defined as severe AKI. The ICU mortality rate in the whole cohort with or without AKI was 48 (9.4%; 95% CI 6.9–12.0%) during the ICU stay. None of the patients had any known congenital abnormality of the kidney. One patient received aminoglycosides (oral streptomycin) during the ICU stay. A comparison of the demographic and clinical characteristics between survivors and non-survivors is displayed in Table 1.

**Table 1:** Comparison of demographic and clinical characteristics between survivors and non-survivors

	Survivors, *n* = 462	Non-survivors, *n* = 48	*P* value
Age, months	1.37 [0.33–16.0]	0.97 [0.33–13.5]	0.540
Age group, *n*			
≤ 28 days, *n* = 239	215 (46.5)	24 (50.0)	0.893
≤ 1 year, *n* = 132	120 (26.0)	12 (25.0)	
> 1 year, *n* = 139	127 (27.5)	12 (25.0)	
Body weight, kg	4.2 [2.5–11.0]	2.9 [1.5–10.0]	0.035
Male, *n*	277 (60.0)	27 (56.3)	0.645
Illness severity^a^, score	6 [3–9]	10 [6–14]	<0.001
Diagnosis on ICU admission			
Respiratory diseases, *n*	176 (38.1)	10 (20.8)	0.054
Neurologic diseases, *n*	59 (12.8)	1 (2.1)	
Preterm/LBW, *n*	37 (8.0)	5 (10.4)	
Sepsis, *n*	45 (9.7)	11 (22.9)	
Poison/trauma/accident, *n*	34 (7.4)	5 (10.4)	
Cardiologic diseases, *n*	20 (4.3)	1 (2.1)	
Gastroenterologic, *n*	19 (4.1)	0 (0)	
Hematologic diseases, *n*	17 (3.7)	6 (12.5)	
MV^b^, *n*	127 (27.5)	35 (72.9)	<0.001
MV duration, days	0 [0–0.84]	2.23 [0–6.86]	<0.001
MV duration ≥ 48 hours, *n*	89 (19.3)	24 (50.0)	<0.001
AKI^c^, *n*	63 (13.6)	16 (33.3)	0.002
AKI stage 1, *n*	36 (7.8)	7 (14.6)	<0.001
AKI stage 2, *n*	19 (4.1)	5 (10.4)	
AKI stage 3, *n*	8 (1.7)	4 (8.3)	
Sepsis^b^, *n*	58 (9.7)	15 (22.9)	0.001
Shock/DIC^b^, *n*	61 (13.2)	13 (27.1)	0.013
MODS^b^, *n*	65 (14.1)	14 (29.2)	0.009
Furosemide^b^, *n*	126 (27.3)	24 (50.0)	0.002
Steroid^b^, *n*	150 (32.5)	21 (43.8)	0.147
Antibiotics^b^, *n*	418 (90.5)	46 (95.8)	0.294
Vancomycin^b^, *n*	35 (7.6)	7 (14.6)	0.099
Mannitol^b^, *n*	115 (24.9)	16 (33.3)	0.225
Inotrope^b^, *n*	60 (13.0)	10 (20.8)	0.182
Hemofiltration^b^, *n*	15 (3.2)	3 (6.3)	0.235
Initial uCysC, ng/mg uCr	311.82 [122.22–975.34]	1587.39 [583.19–8749.16]	<0.001
Peak uCysC, ng/mg uCr	426.49 [165.23–1607.62]	7026.73 [1285.25–33879.56]	<0.001

Values are median [interquartile range] or number (percentage)

*AKI* acute kidney injury, *DIC* disseminated intravascular coagulation, *ICU* intensive care unit, *LB*W low birth weight, *MODS* multi-organ dysfunction syndrome, *MV* mechanical ventilation, *uCysC* urinary cystatin C, *uCr* urinary creatinine

^a^Illness severity was assessed by the score for neonatal acute physiology in critically ill neonates and the pediatric risk of mortality III score in critically ill children

^b^Administered or developed during ICU stay

^c^Developed during the first week after ICU admission

### Urinary CysC

Of the 510 patients, 120 (23.5%) had one sample, 139 (27.3%) had two samples, and 251 (49.2%) had three samples available during the first week after ICU admission. The missing samples were because of patients being discharged from the ICU or death and failure to collect urine. The urinary CysC (uCysC) levels were detectable in 1083 samples (94.1%). Samples with undetectable uCysC were assigned a uCysC value of 5 ng/mL, which is equivalent to half of the detection limit of the assay, to facilitate the calculation of uCysC/uCr ratios. The initial and the peak values of uCysC were used for analysis of association. For each patient, the level of CysC from the urinary sample collected on the first day of admission to ICU was denoted as the initial (first) uCysC. The highest uCysC level among all collected samples was denoted the peak uCysC.

### Correlation between urinary CysC level and clinical variables

There was significant correlation between the initial and peak levels of uCysC and age, body weight, illness severity, duration of MV, AKI stage, sepsis, MODS, and the use of vancomycin, assessed using Spearman’s correlation analysis, as shown in Table 2. Furthermore, variables with a *P* value <0.1 (shown in Table 2) were entered in stepwise multiple linear regression analysis to investigate factors potentially associated with the uCysC levels. As shown in Table 3, illness severity, body weight, AKI stage, MODS, and age were identified as independent factors significantly associated with the initial uCysC levels (total *R*^2^ = 0.25). The levels of peak uCysC were independently associated with illness severity, body weight, MODS, AKI stage, age, and duration of MV (total *R*^2^ = 0.35).

**Table 2:** Spearman’s analysis of correlation between urinary cystatin C and clinical variables (*n* = 510)

	Initial urinary cystatin C		Peak urinary cystatin C	
	Spearman’s *r*	*P* value	Spearman’s *r*	*P* value
Age, months	-0.230	<0.001	-0.307	<0.001
Body weight, kg	-0.302	<0.001	-0.389	<0.001
Male, *n*	-0.005	0.904	-0.024	0.589
Illness severity^a^, score	0.358	<0.001	0.415	<0.001
MV^b^, *n*	0.087	0.051	0.128	0.004
Duration of MV, days	0.093	0.035	0.141	0.001
AKI^c^, *n*	0.172	<0.001	0.164	<0.001
AKI stage	0.182	<0.001	0.175	<0.001
Severe AKI^d^, *n*	0.197	<0.001	0.196	<0.001
Sepsis^b^, *n*	0.040	0.362	0.065	0.141
Shock/DIC^b^, *n*	0.060	0.173	0.076	0.086
MODS^b^, *n*	0.102	0.021	0.122	0.006
Furosemide^b^, *n*	0.070	0.112	0.127	0.004
Steroid^b^, *n*	-0.020	0.655	-0.005	0.919
Antibiotics^b^, *n*	0.080	0.072	0.106	0.017
Vancomycin^b^, n	0.106	0.016	0.121	0.006
Mannitol^b^, *n*	-0.041	0.350	-0.070	0.112
Inotrope^b^, *n*	0.023	0.606	0.034	0.444
Hemofiltration^b^, *n*	0.007	0.880	-0.006	0.898

*r* = Spearman’s correlation coefficient

*AKI* acute kidney injury, *DIC* disseminated intravascular coagulation, *ICU* intensive care unit, *MODS* multi-organ dysfunction syndrome, *MV* mechanical ventilation

^a^Illness severity was assessed by the score for neonatal acute physiology in critically ill neonates and the pediatric risk of mortality III score in critically ill children

^b^Administered or developed during ICU stay

^c^Developed during the first week after ICU admission

^d^Severe AKI was defined as Kidney Disease: Improving Global Outcomes (KDIGO) stages 2 and 3

**Table 3:** Clinical variables potentially associated with urinary cystatin C levels (*n* = 510)*

	Initial urinary cystatin C		Peak urinary cystatin C	
	*B* coefficient (SE)	*P* value	*B* coefficient (SE)	*P* value
Age, months	0.160 (0.059)	0.007	0.159 (0.059)	0.008
Body weight, kg	-0.967 (0.185)	<0.001	-1.174 (0.186)	<0.001
Illness severity^a^, score	0.039 (0.006)	<0.001	0.047 (0.006)	<0.001
AKI stage^b^	0.234 (0.049)	<0.001	0.223 (0.049)	<0.001
MODS^c^	0.327 (0.093)	<0.001	0.368 (0.094)	<0.001
Duration of MV^c^, days	N/A		0.013 (0.005)	0.015

*Variables with a *P* value <0.1 (shown in Table 2) were entered into the stepwise multivariate linear regression analysis. Continuous variables were log-transformed

*MODS* multi-organ dysfunction syndrome, *MV* mechanical ventilation, N/A not applicable

^a^Illness severity was assessed by the score for neonatal acute physiology in critically ill neonates and the pediatric risk of mortality III score in critically ill children

^b^Developed during the first week after ICU admission

^c^Administered or developed during ICU stay

### Association between urinary CysC and AKI

To identify whether uCysC was independently associated with AKI that developed during the first week after ICU admission in critically ill neonates and children, the variables in Table 1 were analyzed by logistic regression. As shown in Table 4, both the initial and peak values of uCysC were significantly associated with AKI. The association between initial (adjusted OR (AOR) = 1.12 per 10,000 ng/mg uCr increase, 95% CI 1.02–1.23, *P* = 0.024) and peak (AOR = 1.11 per 10,000 ng/mg uCr increase, 95% CI 1.02–1.21, *P* = 0.014) uCysC with AKI remained significant after adjustment for age, body weight, illness severity, and MV. A comparison of the initial and the peak values of uCysC among patients grouped according to AKI stage is displayed in Additional file 1: Table S1.

**Table 4:** Association between urinary cystatin C or clinical variables and AKI that developed during the first week after ICU admission (*n* = 510)

	OR	95% CI	*P* value	AOR	95% CI	*P* value
Age, months	1.01	1.00–1.01	0.001	1.00^d^	0.98–1.01	0.592
Body weight, kg	1.04	1.02–1.06	<0.001	1.06^e^	0.99–1.13	0.123
Illness severity^a^, score	1.12	1.07–1.16	<0.001	1.11^f^	1.06–1.16	<0.001
MV^b^	3.13	1.92–5.12	<0.001	2.02^g^	1.16–3.51	0.013
MV duration, days	1.05	1.02–1.08	0.002	1.02^h^	0.98–1.06	0.292
Shock/DIC^b^	2.81	1.59–4.98	<0.001	1.84^h^	0.98–3.44	0.058
MODS^b^	3.50	2.02–6.07	<0.001	1.87^h^	0.99–3.51	0.053
Furosemide^b^	3.85	2.35–6.32	<0.001	2.84^h^	1.62–4.98	<0.001
Steroid^b^	1.84	1.13–2.99	0.015	1.07^h^	0.60–1.91	0.821
Initial uCysC, ng/mg uCr	1.18^c^	1.07–1.31	0.001	1.12^c, h^	1.02–1.23	0.024
Peak uCysC, ng/mg uCr	1.18^c^	1.09–1.28	<0.001	1.11^c, h^	1.02–1.21	0.014

*AKI* acute kidney injury, *AOR* adjusted OR, *CI* confidence interval, *DIC* disseminated intravascular coagulation, *ICU* intensive care unit, *MODS* multi-organ dysfunction syndrome, *MV* mechanical ventilation, *OR* odds ratio, *uCysC* urinary cystatin C, *uCr* urinary creatinine

^a^Illness severity was assessed by the score for neonatal acute physiology in critically ill neonates and the pediatric risk of mortality III score in critically ill children

^b^Administered or developed during ICU stay

^c^Odds ratio represents the increase in risk per 10,000 ng/mg increase in uCysC/uCr

^d^After adjustment for body weight and illness severity

^e^After adjustment for age and illness severity

^f^After adjustment for age and body weight

^g^After adjustment for age, body weight, and illness severity

^h^After adjustment for age, body weight, illness severity, and mechanical ventilation

**Fig. 1:**
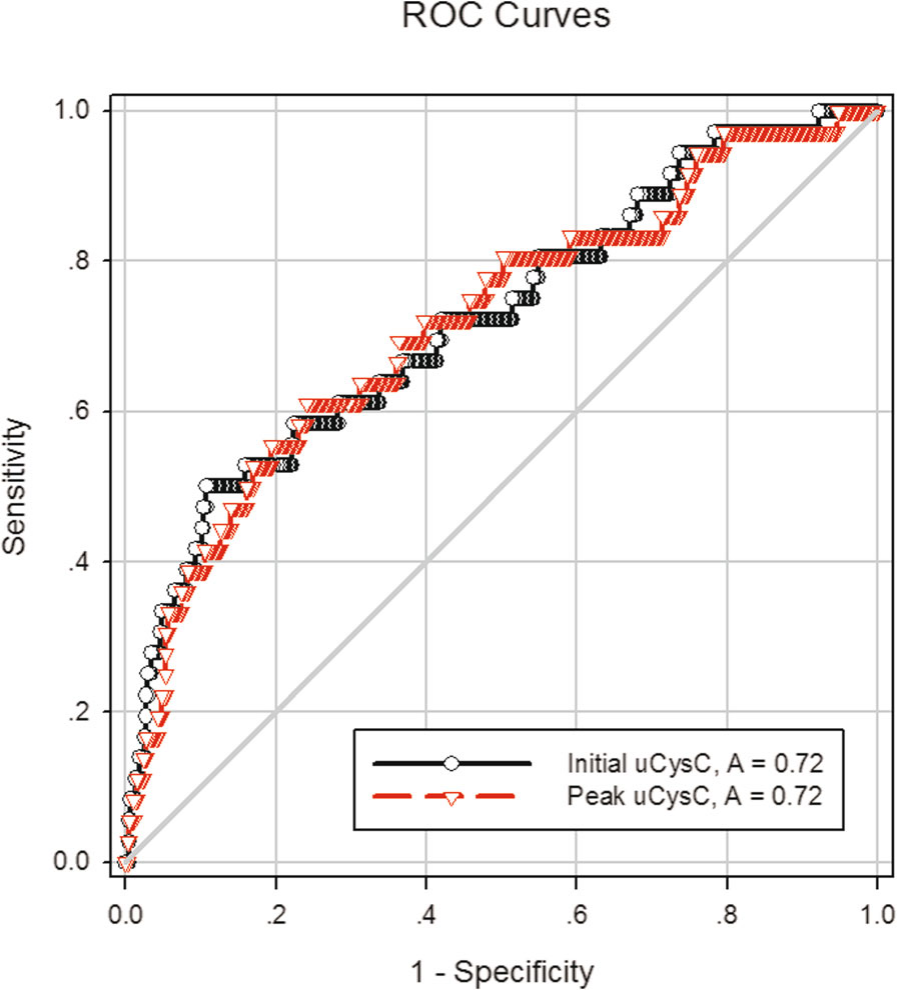
ROC curves for the ability of the initial and the peak urinary cystatin C (uCysC) to predict severe acute kidney injury (AKI) in critically ill neonates and children (*n* = 510). Severe AKI was defined as Kidney Disease: Improving Global Outcomes (KDIGO) stages 2 and 3

The performance of uCysC in predicting AKI and severe AKI is displayed in Table 5. The initial and peak values of uCysC were predictive of severe AKI with an AUC of 0.72 for predicting severe AKI. The ROC curves of the initial and peak uCysC for predicting severe AKI are displayed in Fig. 1. Peak uCysC had sensitivity of 61.1% and specificity of 76.0% at the optimal cutoff value of 1736 ng/mg uCr for predicting severe AKI in critically ill neonates and children, as shown in Table 5.

**Table 5:** Predictive characteristics of urinary cystatin C for AKI or severe AKI (*n* = 510)

uCysC, ng/mg uCr	AUC	95% CI	*P* value	Optimal cutoff value	Sensitivity	Specificity	LR+	LR-
AKI								
Initial uCysC	0.64	0.57–0.71	<0.001	1788.0	38.0%	85.2%	2.6	0.73
Peak uCysC	0.63	0.56–0.70	<0.001	8816.0	30.4%	93.3%	4.5	0.75
Severe AKI								
Initial uCysC	0.72	0.63–0.82	<0.001	3389.0	50.0%	89.1%	4.6	0.56
Peak uCysC	0.72	0.63–0.81	<0.001	1736.0	61.1%	76.0%	2.5	0.51

*AKI* acute kidney injury, *AUC* the area under the ROC curve, *CI* confidence interval, *LR*+ positive likelihood ratio, *LR-* negative likelihood ratio, *uCysC* urinary cystatin C

AKI developed during the first week after ICU admission. Kidney Disease: Improving Global Outcomes (KDIGO) stage 2 or 3 was defined as severe AKI

### Association between urinary CysC and ICU mortality

To identify whether uCysC was independently associated with increased risk of death in critically ill neonates and children, variables with a *P* value <0.05 (shown in the comparison in Table 1), which were considered confounding factors, were analyzed by univariate and multivariate logistic regression. Univariate analysis identified that illness severity, the use and duration of MV, AKI diagnosis and stage, sepsis, shock/DIC, MODS, the use of furosemide, and the initial and peak values of uCysC were significantly associated with mortality. The odds ratio for predicting morality is shown in Table 6. The association between the initial (AOR = 1.13 per 10,000 ng/mg uCr increase, 95% CI 1.01–1.26, *P* = 0.041) and the peak (AOR = 1.17 per 10,000 ng/mg uCr increase, 95% CI 1.07–1.28, *P* < 0.001) values of uCysC and mortality remained significant after adjustment for potential confounders including body weight, illness severity, MV, and AKI stage in multivariate logistic analysis, as displayed in Table 6. A comparison of the mortality rate by quintile of the peak uCysC level is shown in Additional file 2: Figure S1A. The association between uCysC and clinical variables and ICU mortality in critically ill neonates and children, respectively, is shown in Additional file 1: Table S2.

**Table 6:** Association between urinary cystatin C or clinical variables and ICU mortality (*n* = 510)

	OR	95% CI	*P* value	AOR	95% CI	*P* value
Body weight, kg	1.00	0.97–1.03	0.968	0.99^e^	0.95–1.02	0.454
Illness severity^a^, score	1.14	1.09–1.19	<0.001	1.14^f^	1.09–1.20	<0.001
MV^b^	7.10	3.64–13.86	<0.001	4.72^g^	2.28–9.76	<0.001
MV duration, days	1.09	1.05–1.12	<0.001	1.07^g^	1.04–1.11	<0.001
AKI^c^	3.17	1.64–6.10	0.001	2.09^g^	0.94–-3.97	0.049
AKI stage	1.87	1.34–2.60	<0.001	1.49^g^	1.00–4.33	0.031
Severe AKI^c^	3.72	1.63–8.46	0.002	2.25^g^	0.91–5.57	0.079
Sepsis^b^	3.17	1.62–6.18	0.001	2.58^h^	1.22–5.45	0.013
Shock/DIC^b^	2.44	1.22–4.87	0.011	1.32^h^	0.59–2.96	0.500
MODS^b^	2.52	1.28–4.94	0.007	1.18^h^	0.51–2.72	0.706
Furosemide^b^	2.67	1.46–4.87	0.001	1.09^h^	0.53–2.23	0.814
Initial uCysC, ng/mg uCr	1.19^d^	1.08–1.33	0.001	1.13^d, h^	1.01–1.26	0.041
Peak uCysC, ng/mg uCr	1.26^d^	1.16–1.38	<0.001	1.17^d, h^	1.07–1.28	<0.001

*AKI* acute kidney injury, *AOR* adjusted OR, *CI* confidence interval, *ICU* intensive care unit, *DIC* disseminated intravascular coagulation, *MODS*, multi-organ dysfunction syndrome, *MV* mechanical ventilation, *OR* odds ratio, *uCysC* urinary cystatin C, *uCr* urinary creatinine

^a^Illness severity was assessed by the score for neonatal acute physiology in critically ill neonates and the pediatric risk of mortality III score in critically ill children

^b^Administered or developed during ICU stay

^c^Developed during the first week after ICU admission. Severe AKI was defined as Kidney Disease: Improving Global Outcomes (KDIGO) stages 2 and 3

^d^Odds ratio represents the increase in risk per 10,000 ng/mg increase in uCysC/uCr

^e^After adjustment for illness severity

^f^After adjustment for body weight

^g^After adjustment for body weight and illness severity

^h^After adjustment for body weight, illness severity, MV, and AKI stage

**Fig. 2:**
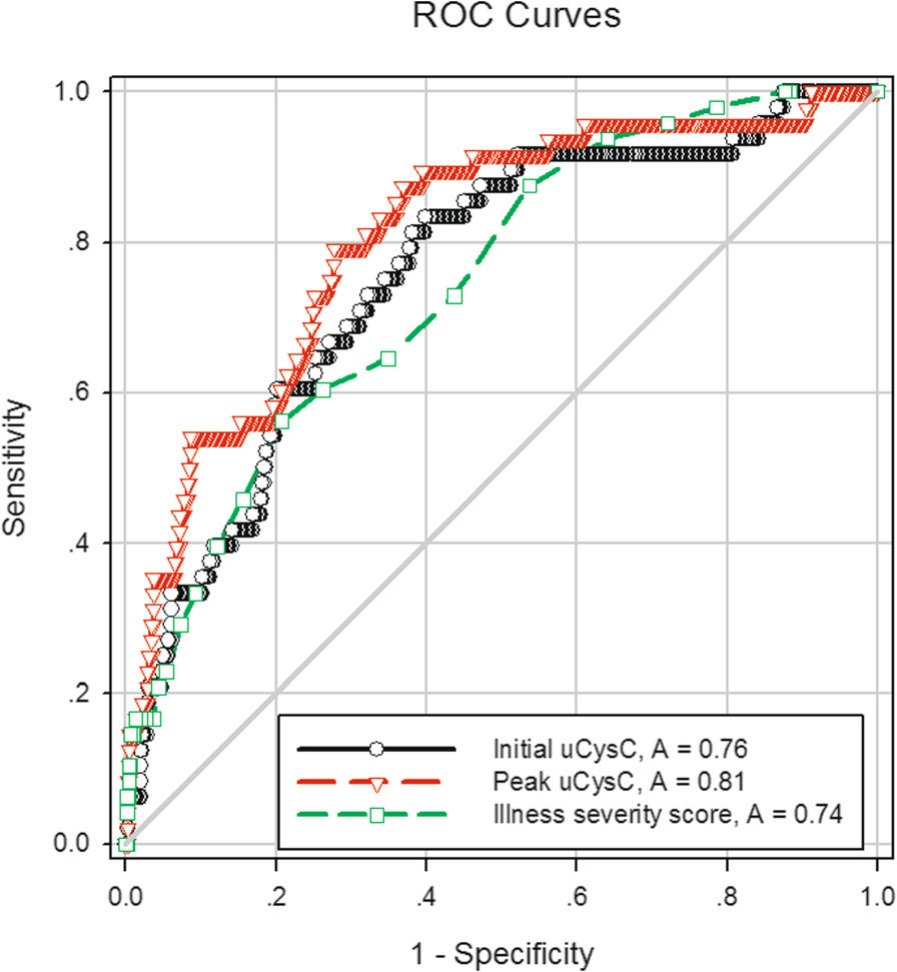
ROC curves for the ability of illness severity score and urinary cystatin C (uCysC) to predict ICU mortality in critically ill neonates and children (*n* = 510). Illness severity was assessed by the score for neonatal acute physiology in critically ill neonates and the pediatric risk of mortality III score in critically ill children

The performance of uCysC in predicting mortality is assessed in Table 7. Both initial and peak values of uCysC predicted mortality. Peak uCysC (AUC = 0.81, P < 0.001) was better than initial uCysC (AUC = 0.76, *P* < 0.001) or illness severity score (AUC = 0.74, *P* < 0.001) in predicting mortality. However, the difference was not statistically significant. The *P* value for the comparison of the AUCs between peak uCysC and illness severity score and between peak and initial uCysC was 0.161 and 0.273, respectively. The ROC curves of uCysC and illness severity score for predicting the mortality of patients are displayed in Fig. 2. We also calculated the optimal cutoff values of uCysC to predict mortality. Peak uCysC had sensitivity of 79.2% and specificity of 72.3% at the optimal cutoff value of 1260 ng/mg uCr in critically ill neonates and children (Table 7). The predictive characteristics of uCysC at different cutoff values for ICU mortality in critically ill neonates and children are displayed in Additional file 1: Table S3. The performance of uCysC in predicting ICU mortality in critically ill neonates and children, respectively, is displayed in Additional file 1: Table S4.

**Table 7:** Performance of urinary cystatin C and clinical variables for prediction of ICU mortality (*n* = 510)

	AUC	95% CI	*P* value	Optimal cutoff value	Sensitivity	Specificity	LR+	LR-
Illness severity^a^, score	0.74	0.68–0.81	<0.001	9.5	56.3%	79.2%	2.7	0.55
MV^b^	0.73	0.65–0.80	<0.001	N/A				
AKI stage^c^	0.60	0.50–0.69	0.020	N/A				
Sepsis^b^	0.59	0.50–0.69	0.033	N/A				
Initial uCysC, ng/mg uCr	0.76	0.69–0.83	<0.001	471.5	83.3%	60.2%	2.1	0.28
Peak uCysC, ng/mg uCr	0.81	0.75–0.88	<0.001	1260.0	79.2%	72.3%	2.9	0.29

*AKI* acute kidney injury, *AUC* the area under the ROC curve, *CI* confidence interval, *ICU* intensive care unit, *LR+* likelihood ratio positive, *LR-* likelihood ratio negative, *MV* mechanical ventilation, *N/A* not applicable, *uCysC* urinary cystatin C, *uCr* urinary creatinine

^a^Illness severity was assessed by the score for neonatal acute physiology in critically ill neonates and the pediatric risk of mortality III score in critically ill children

^b^Administered or developed during ICU stay

^c^Developed during the first week after ICU admission

### Association between urinary CysC and secondary outcomes

Both the initial and the peak values of uCysC were associated with the duration of MV (initial, unstandardized coefficient *Β* = 0.202, SE = 0.069, *P* = 0.003; peak, *B* = 0.277, SE = 0.064, *P* < 0.001) and the length of the ICU stay (initial, *B* = 0.159, SE = 0.028, *P* < 0.001; peak, *B* = 0.231, SE = 0.025, *P* < 0.001) by using univariate linear regression analysis. The peak uCysC, but not the initial uCysC, remained significantly associated with the duration of MV (*B* = 0.183, SE = 0.071, *P* = 0.010) and the length of the ICU stay (*B* = 0.110, SE = 0.025, *P* < 0.001) after adjustment for body weight and illness severity in multivariate linear regression analysis. Univariate and multivariate linear regression analyses were performed with log-transformed data on the duration of MV and length of the ICU stay. Patients who did not receive MV were arbitrarily given a value of -2. A comparison of the length of the ICU stay by quintile of the peak uCysC level is shown in Additional file 2: Figure S1B.

### Association between urinary CysC-positive subclinical AKI and ICU mortality

To identify whether uCysC-positive subclinical AKI that developed during the first week after admission was associated with increased risk of adverse outcomes in patients, “uCysC(−)” and “uCysC(+)”, indicating the absence and presence of tubular injury, were defined by the optimal cutoff value of peak uCysC (1260 ng/mg uCr) for predicting mortality. Patients were classified as follows: uCysC(−)/AKI(−), uCysC(+)/AKI(−), uCysC(−)/AKI(+), and uCysC(+)/AKI(+). The clinical characteristics and outcomes grouped according to these four groups are shown in Table 8. Among 510 patients, 25.5% developed uCysC(+)/AKI(−) status, which was much more common than uCysC(−)/AKI(+). Mortality in patients with uCysC(+)/AKI(−) status was significantly higher than that in patients with uCysC(−)/AKI(−). The difference remained significant after adjusting for body weight and illness severity (*P* < 0.001). Although mortality in patients with uCysC(+)/AKI(−) tended to be higher than that in patients with uCysC(−)/AKI(+) status, the difference did not reach significant (*P* = 0.162). There was also no significant difference in mortality between patients with uCysC(+)/AKI(−) and those with uCysC(+)/AKI(+) (*P* = 0.116). Mortality was significantly higher in patients positive for both uCysC(+)/AKI(+) than in patients with uCysC(−)/AKI(−) (*P* < 0.001) and in patients with uCysC(−)/AKI(+) status (*P* = 0.011). However, the difference between uCysC(+)/AKI(+) and uCysC(−)/AKI(+) did not remain significant after controlling for body weight and illness severity (*P* = 0.077), as shown in Table 8. In addition, the first uCysC level was below the cutoff value of 1260 ng/mg uCr in 388 of 510 patients. Among the 388 patients, 48 developed uCysC(+) status with or without AKI, during serial measurements. These patients had significantly higher mortality rates (18.8%) compared to those with uCysC(−) status with or without AKI during the first week after ICU admission (3.1%, *P* = 0.001). Demographic and clinical characteristics and outcomes grouped according to uCysC and AKI status in critically ill neonates and children, respectively, are shown in Additional file 1: Tables S5-S6.

**Table 8:** Demographic and clinical characteristics and outcomes grouped according to urinary cystatin C and AKI status

	uCysC(−)/ AKI(−)	uCysC(+)/ AKI(−)	uCysC(−)/ AKI(+)	uCysC(+)/ AKI(+)	*P* value
Number	301 (59.0)	130 (25.5)	43 (8.4)	36 (7.1)	N/A
Body weight, kg	5.5 [3.0–12.0]	2.4 [1.5–5.0]^*^	10.0 [3.4–19.0]^*#^	5.0 [1.4–13.0]^#^	<0.001
Male, *n*	184 (61.1)	68 (52.3)	28 (65.1)	24 (66.7)	0.216
Illness severity^a^, score	5 [2–7]	8 [5–10]^*^	7 [5–11]^*^	12 [8–16.5]^*#&^	<0.001
MV, *n*	77 (25.6)	42 (32.3)	19 (44.2)^*^	24 (66.7)^*#^	<0.001
MV duration, days	0 [0–0.38]	0 [0–1.10]	0 [0–3.96]^*^	3.16 [0–5.65]^*#&^	<0.001
Severe AKI^b^, *n*	0 (0)	0 (0)	14 (32.6)^*#^	22 (61.1)^*#&^	<0.001
ICU LOS, hours	121.0 [56.0–228.1]	336.0 [132.9–774.0]^*^	144.0 [63.0–288.0]^#^	236.0 [137.0–917.8]^*&^	<0.001
Death, *n*	6 (2.0)	26 (20.0)^*^	4 (9.3)^*^	12 (33.3)^*&^	<0.001^c^

Values are median [interquartile range] or number (percentage). uCysC(−) indicates the absence of tubular injury, and uCysC(+) indicates the presence of tubular injury defined by the optimal cutoff value of the peak uCysC for predicting mortality (1260 ng/mg uCr)

*AKI* acute kidney injury, *ICU*, intensive care unit, *LOS* length of stay, *MV* mechanical ventilation, *uCysC* urinary cystatin C, *uCr* urinary creatinine

^a^Illness severity was assessed by the score for neonatal acute physiology in critically ill neonates and the pediatric risk of mortality III score in critically ill children

^b^Severe AKI was defined as Kidney Disease: Improving Global Outcomes (KDIGO) stages 2 and 3

^c^*P* = 0.001, after adjustment for body weight, illness severity, MV, and severe AKI

^*^P < 0.05 vs. uCysC(−)/AKI(−). ^#^*P* < 0.05 vs. uCysC(+)/AKI(−). ^&^*P* < 0.05 vs. uCysC(−)/AKI(+)

To strengthen the conclusion of association between uCysC-positive subclinical AKI and ICU mortality, the uCysC(+)/AKI(−) population was further defined based on different cutoff values of uCysC. Comparisons of mortality rates among the four groups of patients grouped by uCysC/AKI status (defined by the optimal cutoff value of the initial or peak uCysC for predicting AKI, severe AKI, or mortality) in critically ill neonates and children are shown in Additional file 1: Table S7. Demographic and clinical characteristics and outcomes grouped according to uCysC/AKI status, which was defined by the optimal cutoff value of the initial uCysC (1788 ng/mg uCr) for predicting AKI, are displayed in Additional file 1: Table S8.

Furthermore, the analysis of association between uCysC-positive subclinical AKI and mortality is shown in Table 9. The OR for patients with uCysC(+)/AKI(−) status having an increased risk of mortality compared to those with uCysC(−)/AKI(−) status was 12.29 (95% CI, 4.92–30.70; *P* < 0.001). The association remained significant on multivariate logistic regression analysis after adjusting for body weight and illness severity (AOR = 9.34, 95% CI 3.55–24.61, *P* < 0.001), as shown in Table 9.

**Table 9:** Association between urinary cystatin C-positive subclinical AKI and adverse outcomes

Outcomes	uCysC(+)/AKI(−) vs. uCysC(−)/AKI(−)
Mortality, *n*	OR 12.29 (95% CI 4.92–30.70), *P* < 0.001
	AOR^b^ 9.34 (95% CI 3.55–24.61), *P* < 0.001
Length of ICU stay^a^, hours	*B* 0.392 (SE 0.050), *P* < 0.001
	*B*^c^ 0.111 (SE 0.045), *P* = 0.015

B is the unstandardized coefficient

*AKI* acute kidney injury, *AOR* adjusted OR, *CI* confidence interval, *ICU* intensive care unit, *OR* odds ratio, *SE* standard error, *uCysC* urinary cystatin C

^a^ICU length of stay was log-transformed for linear regression analysis

^b^After adjustment for body weight and illness severity using multivariate binary logistic regression analysis

^c^After adjustment for body weight and illness severity using multivariate linear regression analysis; body weight was log-transformed due to skewed distribution

### Association between urinary CysC-positive subclinical AKI and secondary outcomes

There was no significant difference in the duration of MV between patients with uCysC−/AKI− and uCysC+/AKI− (P = 0.178) status. Compared to patients negative for both (uCysC−/AKI−), patients with uCysC(+)/AKI(−) spent 2.8 times as long in the ICU (*P* < 0.001). Univariate linear regression analysis further revealed that uCysC+/AKI− status was significantly associated with a longer ICU stay, compared to uCysC−/AKI− status (*B* = 0.392, SE = 0.050, *P* < 0.001). The association remained significant after adjustment for body weight and illness severity (*B* = 0.111, SE = 0.045, *P* = 0.015) using multivariate linear regression analysis, as shown in Table 9.

## Discussion

Our data indicate that subclinical AKI may occur without detectable loss of kidney function and predict worse clinical outcomes, and that urinary CysC is capable of detecting subclinical AKI in critically ill neonates and children.

Urinary CysC was associated with AKI in critically ill neonates and children in this study. The findings further strengthen the evidence for a relationship between elevated uCysC and AKI [11, 19, 20, 30]. Clinical studies investigating the association between uCysC and mortality have been limited mainly to adult patients [18, 19]. Studies in general critically ill adults demonstrate that uCysC is an independent predictor of mortality [18, 19]. In infants undergoing cardiac surgery with cardiopulmonary bypass, elevated uCysC in the early postoperative period is associated with a greater risk of death or need for renal replacement therapy [31]. Compared to the previous study in infants undergoing cardiac surgery, our study included a general NICU and PICU population. Nevertheless, our results are in line with the previous study and indicate that uCysC is a sensitive biomarker for predicting mortality in critically ill neonates and children. For every 1% increase in uCysC (10,000 ng/mg uCr), the odds of mortality increased by 26%. We further demonstrated that the association between uCysC and mortality was independent of AKI and illness severity. Urinary CysC-positive critically ill neonates and children, with or without clinical AKI diagnosed by the KDIGO criteria, developed worse clinical outcomes.

The major finding in this study was that 25.5% of patients who had an increased risk of adverse outcomes were identified based on increased uCysC, in the absence of oliguria or increased sCr (uCysC+/AKI−). It is well-known that increased sCr is not detected until the glomerular filtration rate is reduced by > 50% [15, 32]. AKI can be diagnosed early by damage or functional biomarkers preceding loss of filtration function [12, 13, 15, 33]. Increased uCysC has been demonstrated to reflect renal tubular injury and impairment and predict AKI 24–48 h before sCr [11]. Our data indicate that this substantial group of critically ill patients with positive uCysC who do not fulfill current KIDGO criteria for AKI are likely to have subclinical AKI, and uCysC can detect subclinical AKI when tubular damage has occurred without oliguria or appreciable increase in sCr.

To our knowledge, this study is the first prospective investigation to determine the association between subclinical AKI and clinical outcomes in critically ill patients. The development of subclinical AKI in critically ill neonates and children is associated with negative clinical outcomes. Our data agree with a previous report conducted in adult patients with cardiorenal syndrome type 1, which was a retrospective analysis of pooled data from ten prospective observational studies of neutrophil gelatinase-associated lipocalin (NGAL) and demonstrated that in the absence of a diagnostic increases in sCr, NGAL can be used to identify patients with subclinical AKI who have an increased risk of adverse outcomes [15]. It is known that sCr fails to identify likely AKI in some patients who are at an increased risk of death [9, 15]. The patients with uCysC-positive subclinical AKI were at greater risk of longer ICU stay and mortality compared to those with uCysC(−)/AKI(−), suggesting that the concept of subclinical AKI as a diagnosis with promise for improving the management of AKI is clinically relevant and that critically ill neonates and children with subclinical AKI might benefit from early diagnosis and treatment.

In this study, we also found that 8.4% of the patients had no biomarker evidence of tubular injury but had loss of kidney function (uCysC−/AKI+), suggesting that this subgroup of patients who had functional change without damage might have pre-renal azotemia, which is considered a volume-responsive, reversible alteration in kidney function [8, 34]. These patients had a fourfold increased risk of mortality compared to patients negative for both (uCysC−/AKI−). A further study is warranted to explore whether the combined use of uCysC with sCr and/or urine output might differentiate between AKI and pre-renal azotemia.

Moreover, the patients with uCysC(+)/AKI(+) had the greatest risk of mortality, which is consistent with findings from the multicenter pooled analysis of the adult studies [15] and supports the concept that subclinical tubular injury precedes detectable decreased kidney function and that when both features occur together, clinical outcomes are worse. Interestingly, our data confirm the potential classification of patients according to the conceptual framework based on a combination of damage and functional biomarkers. The framework is proposed by the Acute Dialysis Quality Initiative (ADQI) conference and provides a potent approach for assessing patients with AKI for diagnosis, staging, differential diagnosis, and prognosis in order to enable clinicians and researchers to most effectively utilize AKI biomarkers [8].

There are a number of limitations to our study. First, the study was not a multicenter study. Nevertheless, the relatively large sample size, with a prospective study design and a protocol with frequent uCysC measurements, provided adequate power for assessing the association between uCysC and mortality. Second, the diagnosis of neonatal AKI based on sCr and urine output is troublesome [27, 35–37]. Serum Cr reflects maternal levels during the first 2–3 days of life and declines progressively with increasing postnatal age to reach a stable neonatal level by 2 weeks of life in neonates with more than 26 weeks of gestational age [38, 39]. The total body water content and the urine output in neonates are normally greater than those in other populations, suggesting that urine output ≤ 0.5 mL/kg/h is a nonsensitive marker of neonatal AKI [37]. Therefore, the diagnosis and staging of AKI based on neonatal AKI KDIGO may have underestimated the incidence and grade of AKI in neonates [1, 40]. Moreover, the results from the NICU population seem to be a large driver of the overall study results, which would bias the study towards positive results. Nevertheless, the PICU analyses strengthen the fact that there truly is a signal in the results because the older children do not have this issue to the same extent but still seem to have uCysC-positive subclinical AKI that increases mortality risk. Third, the majority of critically ill children did not have baseline sCr measurement within 3 months prior to PICU admission. The incidence of AKI may be underestimated when sCr at PICU admission is used as a baseline; therefore, the lowest sCr within 2 weeks in the PICU was used as a baseline for patients with elevated sCr ≥ 1.2 mg/dL (106.1 μmol/L) at admission. Although it has not been validated in critically ill children, a previous study suggests that AKI incidence is best estimated by choosing the lowest sCr value within the first week in the ICU as a baseline sCr [29]. Fourth, we did not measure uCysC daily as part of the study, which may underestimate the incidence and grade of AKI and limit our ability to determine the exact time at which the levels of uCysC rise prior to a sCr increase. Fifth, there is no specific cutoff value for uCysC to identify AKI and mortality in critically ill neonates and children. We set the uCysC level of 1260 ng/mg uCr, which was the optimal cutoff value for uCysC to predict mortality, as the threshold for identifying tubular injury. The discrepancy in cutoff points between this study and our previous study [20] or between critically ill neonates and children in this study may be related to the fact that we evaluated the predictive accuracy of uCysC in a general and heterogeneous NICU and PICU population in this study. The level of urinary CysC was significantly affected by postnatal age [41]. Sixth, the concept of uCysC-positive subclinical AKI has not been validated in different case mixes, especially in patients with higher scores of illness severity. Further multicenter prospective studies are necessary to confirm our findings and define age-related reference values of uCysC for neonates and children.

## Conclusions

Both the initial and the peak levels of uCysC are significantly associated with AKI and mortality and are independently predictive of ICU mortality in critically ill neonates and children. Subclinical AKI may occur without detectable loss of kidney function. Urinary level of CysC is a sensitive detector of subclinical AKI, and uCysC-positive subclinical AKI is associated with worse clinical outcomes in critically ill neonates and children. Further randomized and controlled clinical trials are needed to explore whether treatment of subclinical AKI would improve the clinical outcomes in this population.

## Additional files

Additional file 1: **Table S1.** Urinary cystatin C levels grouped according to AKI status. **Table S2.** Association of urinary cystatin C and clinical variables with ICU mortality in critically ill neonates and children, respectively**. Table S3.** Predictive characteristics of urinary cystatin C at different cutoff values for ICU mortality in critically ill neonates and children. **Table S4.** Predictive characteristics of urinary cystatin C for ICU mortality in critically ill neonates and children, respectively. **Table S5.** Demographic and clinical characteristics and outcomes grouped according to urinary cystatin C and AKI status in critically ill neonates. **Table S6.** Demographic and clinical characteristics and outcomes grouped according to urinary cystatin C and AKI status in critically ill children. **Table S7.** Comparison of mortality rates among groups of uCysC/AKI status defined by different cutoff values of urinary cystatin C. **Table S8.** Demographic and clinical characteristics and outcomes grouped according to urinary cystatin C and AKI status defined by the optimal cutoff value of the initial uCysC for predicting AKI. (DOCX 53 kb)

Additional file 2: **Figure S1.** Comparison of ICU mortality (A) and the length of ICU stay (B) by quintile of peak value of urinary cystatin C in critically ill neonates and children (*n* = 510). The peak values of urinary CysC were divided into quintiles, *n* = 102 in each group. *P* value refers to comparison among five groups. ^*^P < 0.05 vs. the first; ^#^*P* < 0.05 vs. the second; ^&^*P* < 0.05 vs. the third; ^$^*P* < 0.05 vs. the fourth quintile. **A** Error bars represent the mortality rate and 95% confidence interval. **B** Error bars represent the mean and 95% confidence interval. (TIF 72 kb)

## Abbreviations

AKI: Acute kidney injury
AOR: Adjusted odds ratio
AUC: Area under the receiver operating characteristic curve
CI: Confidence interval
Cr: Creatinine
CysC: Cystatin C
DIC: Disseminated intravascular coagulation
IQR: Interquartile range
KDIGO: Kidney Disease: Improving Global Outcomes
LOS: Length of stay
LR-: Likelihood ratio negative
LR+: Likelihood ratio positive
MODS: Multiple organ dysfunction syndrome
MV: Mechanical ventilation
NGAL: Neutrophil gelatinase-associated lipocalin
NICU: Neonatal intensive care unit
OR: Odds ratio
PICU: Pediatric intensive care unit
PRISM III: Pediatric risk of mortality III
ROC: Receiver operating characteristic curve
sCr: Serum creatinine
SNAP: Score for neonatal acute physiology
uCysC: Urinary cystatin C
