# Supplementary material for: Subclinical acute kidney injury is associated with adverse outcomes in critically ill neonates and children
Source: Crit Care. 2018 Oct 10;22:256. doi: 10.1186/s13054-018-2193-8 (PMC6180629; doi:10.1186/s13054-018-2193-8)
Supplement: Supplementary file 1 — Table S1. Urinary cystatin C levels grouped according to AKI status. Table S2. Association of urinary cystatin C and clinical variables with ICU mortality in critically ill neonates and children, respectively. Table S3. Predictive characteristics of urinary cystatin C at different cutoff values for ICU mortality in critically ill neonates and children. Table S4. Predictive characteristics of urinary cystatin C for ICU mortality in critically ill neonates and children, respectively. Table S5. Demographic and clinical characteristics and outcomes grouped according to urinary cystatin C and AKI status in critically ill neonates. Table S6. Demographic and clinical characteristics and outcomes grouped according to urinary cystatin C and AKI status in critically ill children. Table S7. Comparison of mortality rates among groups of uCysC/AKI status defined by different cutoff values of urinary cystatin C. Table S8. Demographic and clinical characteristics and outcomes grouped according to urinary cystatin C and AKI status defined by the optimal cutoff value of the initial uCysC for predicting AKI. (DOCX 53 kb) [file 13054_2018_2193_MOESM1_ESM.docx]

**Table S1** Urinary cystatin C levels grouped according to AKI status

|  | **n** | **Initial** **uCysC, ng/mg uCr** | **Peak uCysC, ng/mg uCr** |
| --- | --- | --- | --- |
| Non-AKI | 431 | 336.47 [119.99-1024.37] | 474.38 [167.88-1685.82] |
| AKI stage 1 | 43 | 397.77 [163.64-2088.60] | 540.23 [217.39-5672.27] |
| AKI stage 2 | 24 | 1520.43 [180.97-17459.67]^*^ | 2459.26 [235.11-17459.09]^*^ |
| AKI stage 3 | 12 | 3916.35 [601.88-87853.92]^*#^ | 16001.03 [1074.77-121052.62]^*#&^ |
| P |  | <0.001 | <0.001 |

Values are median [interquartile range].

AKI was developed during the first week after admission.

^*^P<0.05 vs. non-AKI; ^#^P<0.05 vs. AKI stage 1; ^&^P<0.05 vs. AKI stage 2.

**Table S2** Association of urinary cystatin C and clinical variables with ICU mortality in critically ill neonates and children, respectively

|  | **Neonates (n=239)** | | | **Children (n=271)** | | |
| --- | --- | --- | --- | --- | --- | --- |
|  | **OR** | **95% CI** | **P** | **OR** | **95% CI** | **P** |
| Age, months | 0.44 | 0.02-110.71 | 0.617 | 1.00 | 0.99-1.01 | 0.540 |
| Body weight, kg | 0.24 | 0.12-0.48 | <0.001 | 1.01 | 0.98-1.05 | 0.495 |
| Gender | 1.11 | 0.48-2.59 | 0.809 | 1.20 | 0.51-2.82 | 0.670 |
| Illness severity, score | 1.14 | 1.01-1.29 | 0.028^d^ | 1.15 | 1.09-1.21 | <0.001^c^ |
| MV | 4.51 | 1.86-10.89 | 0.001^e^ | N/A | N/A | <0.001 |
| MV duration, hours | 1.00^a^ | 0.99-1.01 | 0.113 | 1.11^a^ | 1.06-1.16 | <0.001^e^ |
| AKI | 2.78 | 1.00-7.72 | 0.049^f^ | 3.70 | 1.53-8.91 | 0.004^f^ |
| AKI stage | 1.67 | 1.02-2.75 | 0.041^f^ | 2.10 | 1.33-3.29 | 0.001^f^ |
| Severe AKI | 5.18 | 1.43-18.71 | 0.012^e^ | 3.16 | 1.06-9.40 | 0.039^f^ |
| Furosemide | 1.74 | 0.70-4.31 | 0.232 | 4.33 | 1.78-10.55 | 0.001^h^ |
| Initial uCysC, ng/mg uCr | 1.49^b^ | 1.16-1.91 | 0.002^g^ | 1.08^b^ | 0.95-1.24 | 0.242 |
| Peak uCysC, ng/mg uCr | 1.39^b^ | 1.18-1.64 | <0.001^g^ | 1.20^b^ | 1.09-1.33 | <0.001^h^ |

AKI, acute kidney injury; AOR, Adjusted OR; CI, confidence interval; ICU, intensive care unit; MV, mechanical ventilation; OR, odds ratio.

Illness severity was assessed by the score for neonatal acute physiology in critically ill neonates and the pediatric risk of mortality III score in critically ill children. Severe AKI was defined as KDIGO stages 2 and 3. N/A: none of the patients without MV died during PICU stay (Probability value: Fisher’s exact test).

^a^Odds ratio represents the increase in risk per 24 hours increase in MV duration. ^b^Odds ratio represents the increase in risk per 10,000 ng/mg increase in uCysC/uCr.

^c^P<0.05; ^d^P>0.05 After adjustment for body weight. ^e^P<0.05; ^f^P>0.05 After adjustment for body weight and illness severity. ^g^P<0.05; ^h^P>0.05 After adjustment for body weight, illness severity, MV, and severe AKI.

**Table S3** Predictive characteristics of urinary cystatin C at different cut-off values for ICU mortality in critically ill neonates and children (n=510)

|  | **Cut-off values** | **Sensitivity %** | **Specificity %** | **LR+** | **LR-** |
| --- | --- | --- | --- | --- | --- |
| Initial uCysC/uCr | 471.5 ng/mg | 83.3 | 60.2 | 2.1 | 0.28 |
|  | 1260 ng/mg | 60.4 | 80.0 | 3.0 | 0.50 |
|  | 1736 ng/mg | 43.8 | 82.9 | 2.6 | 0.68 |
|  | 1788 ng/mg | 41.7 | 84.0 | 2.6 | 0.70 |
|  | 2500 ng/mg | 39.6 | 86.4 | 2.9 | 0.70 |
|  | 3389 ng/mg | 35.4 | 88.7 | 3.1 | 0.73 |
|  | 8816 ng/mg | 25.0 | 95.0 | 5.0 | 0.79 |
| Peak uCysC/uCr | 471.5 ng/mg | 91.7 | 52.0 | 1.9 | 0.16 |
|  | 1260 ng/mg | 79.2 | 72.3 | 2.9 | 0.29 |
|  | 1736 ng/mg | 64.6 | 77.3 | 2.8 | 0.46 |
|  | 1788 ng/mg | 62.5 | 78.1 | 2.9 | 0.48 |
|  | 2500 ng/mg | 56.3 | 81.6 | 3.1 | 0.54 |
|  | 3389 ng/mg | 54.1 | 84.9 | 3.6 | 0.54 |
|  | 8816 ng/mg | 41.7 | 92.9 | 5.8 | 0.63 |

ICU, intensive care unit; LR+, positive likelihood ratio; LR-, negative likelihood ratio.

**Table S4** Predictive characteristics of urinary cystatin C for ICU mortality in critically ill neonates and children, respectively

|  | **AUC** | **95% CI** | **P** | **Optimal cut-off value** | **Sensitivity** | **Specificity** | **LR+** | **LR-** |
| --- | --- | --- | --- | --- | --- | --- | --- | --- |
| **Neonates (n=239)** | | | | | | | | |
| Initial uCysC, ng/mg uCr | 0.82 | 0.73-0.91 | <0.001 | 1255.0 | 79.2% | 77.7% | 3.5 | 0.27 |
| Peak uCysC, ng/mg uCr | 0.84 | 0.76-0.93 | <0.001 | 1558.0 | 95.8% | 67.0% | 2.9 | 0.06 |
| **Children (n=271)** | | | | | | | | |
| Initial uCysC, ng/mg uCr | 0.72 | 0.63-0.83 | <0.001 | 281.2 | 87.5% | 58.7% | 2.1 | 0.21 |
| Peak uCysC, ng/mg uCr | 0.81 | 0.73-0.90 | <0.001 | 651.4 | 83.3% | 71.7% | 2.9 | 0.23 |

AUC, the area under the ROC curve; CI, confidence interval; ICU, intensive care unit; LR+, likelihood ratio positive; LR-, likelihood ratio negative.

**Table S5** Demographic and clinical characteristics and outcomes grouped according to urinary cystatin C and AKI status in critically ill neonates (n=239)

|  | **uCysC(−)/ AKI(−)** | **uCysC(+)/ AKI(−)** | **uCysC(−)/ AKI(+)** | **uCysC(+)/ AKI(+)** | **P** |
| --- | --- | --- | --- | --- | --- |
| n | 131 (54.8) | 79 (33.1) | 14 (5.9) | 15 (6.3) | N/A |
| Body weight, kg | 2.9 [2.0-3.3] | 1.7 [1.3-2.4]^*^ | 2.5 [1.5-3.4]^#^ | 1.4 [1.1-2.2]^*&^ | <0.001 |
| Male, n | 76 (58.0) | 38 (48.1) | 11 (78.6) | 10 (66.7) | 0.527 |
| Illness severity, score | 6 [5-8] | 8 [6-10]^*^ | 8 [5-10] | 11 [8-13]^*#^ | <0.001 |
| MV, n | 20 (15.3) | 15 (19.0) | 2 (14.3) | 8 (53.3)^*#&^ | 0.005 |
| MV Duration, hours | 0 [0-0] | 0 [0-0] | 0 [0-0] | 66 [0-156]^*#&^ | 0.001 |
| Severe AKI, n | 0 (0) | 0 (0) | 4 (28.6)^*#^ | 8 (53.3)^*#^ | <0.001 |
| ICU LOS, hours | 216 [144-360] | 480 [264-960]^*^ | 300 [162-420]^#^ | 960 [216-1344]^*^ | <0.001 |
| Death, n | 1 (0.8) | 17 (21.5)^*^ | 0 (0) | 6 (40.0)^*&^ | <0.001^a^ |

Values are median [interquartile range]. Numbers in parentheses denote percentages.

AKI, acute kidney injury; ICU, intensive care unit; LOS, length of stay; MV, mechanical ventilation.

uCysC(−) indicates the absence of tubular injury, and uCysC(+) indicates the presence of tubular injury defined by the optimal cut-off value of the peak uCysC for predicting mortality in critically ill neonates (1558.0 ng/mg uCr).

Illness severity was assessed by the score for neonatal acute physiology in critically ill neonates. Severe AKI was defined as KDIGO stages 2 and 3.

^a^P=0.005 After adjustment for body weight and illness severity.

^*^P<0.05 vs. uCysC(−)/AKI(−). ^#^P<0.05 vs. uCysC(+)/AKI(−). ^&^P<0.05 vs. uCysC(−)/AKI(+).

**Table S6** Demographic and clinical characteristics and outcomes grouped according to urinary cystatin C and AKI status in critically ill children (n=271)

|  | **uCysC(−)/ AKI(−)** | **uCysC(+)/ AKI(−)** | **uCysC(−)/ AKI(+)** | **uCysC(+)/ AKI(+)** | **P** |
| --- | --- | --- | --- | --- | --- |
| n | 154 (56.8) | 67 (24.7) | 27 (9.9) | 23 (8.5) | N/A |
| Body weight, kg | 11.0 [7.0-18.1] | 8.0 [5.0-11.5]^*^ | 15.0 [9.0-23.0]^#^ | 13.0 [7.0-28.0]^#^ | <0.001 |
| Male, n | 99 (64.3) | 39 (58.2) | 16 (59.3) | 15 (65.2) | 0.819 |
| Illness severity, score | 3 [0-6] | 6 [2-11]^*^ | 7 [4-11]^*^ | 14 [9-20]^*#&^ | <0.001 |
| MV, n | 50 (32.5) | 34 (50.7)^*^ | 15 (55.6)^*^ | 18 (78.3)^*#^ | <0.001 |
| MV Duration, hours | 0 [0-44.5] | 0 [0-179]^*^ | 22 [0-123]^*^ | 81.5 [5-138]^*^ | <0.001 |
| Severe AKI, n | 0 (0) | 0 (0) | 8 (29.6)^*#^ | 16 (69.6)^*#&^ | <0.001 |
| ICU LOS, hours | 70.8 [42.0-140.2] | 118.5 [46.0-291.4]^*^ | 71.0 [39.0-167.0] | 168.0 [81.0-377.4]^*^ | 0.002 |
| Death, n | 1 (0.6) | 13 (19.4)^*^ | 3 (11.1)^*^ | 7 (30.4)^*^ | <0.001^a^ |

Values are median [interquartile range]. Numbers in parentheses denote percentages.

AKI, acute kidney injury; ICU, intensive care unit; LOS, length of stay; MV, mechanical ventilation.

uCysC(−) indicates the absence of tubular injury, and uCysC(+) indicates the presence of tubular injury defined by the optimal cut-off value of the peak uCysC for predicting mortality in critically ill children (651.4 ng/mg uCr).

Illness severity was assessed by the pediatric risk of mortality III score in critically ill children. Severe AKI was defined as KDIGO stages 2 and 3.

^a^P=0.011 After adjustment for body weight and illness severity.

^*^P<0.05 vs. uCysC(−)/AKI(−). ^#^P<0.05 vs. uCysC(+)/AKI(−). ^&^P<0.05 vs. uCysC(−)/AKI(+).

**Table S7** Comparison of mortality rates among groups of uCysC/AKI status defined by different cut-off values of urinary cystatin C (n=510)

|  | **Cut-off values** | **uCysC(−)/ AKI(−)** | **uCysC(+)/ AKI(−)** | **uCysC(−)/ AKI(+)** | **uCysC(+)/ AKI(+)** | **P** |
| --- | --- | --- | --- | --- | --- | --- |
| Initial uCysC/uCr | 1788 ng/mg | 23/344 (6.3) | 9/55 (14.1)^*^ | 5/44 (10.2) | 11/19 (36.7)^*#&^ | <0.001 |
|  | 3389 ng/mg | 25/363 (6.4) | 7/36 (16.3)^*^ | 6/47 (11.3) | 10/16 (38.5)^*#&^ | <0.001 |
|  | 471.5 ng/mg | 5/247 (2.0) | 27/152 (15.1)^*^ | 3/31 (8.8) | 13/32 (28.9)^*#&^ | <0.001 |
| Peak uCysC/uCr | 8816 ng/mg | 22/380 (5.5) | 10/19 (34.5)^*^ | 6/49 (10.9)^#^ | 10/14 (41.7)^*&^ | <0.001 |
|  | 1736 ng/mg | 13/317 (3.9) | 19/82 (18.8)^*^ | 4/40 (9.1) | 12/23 (34.3)^*&^ | <0.001 |
|  | 1260 ng/mg | 6/295 (2.0) | 26/104 (20.0)^*^ | 4/39 (9.3)^*^ | 12/24 (33.3)^*&^ | <0.001 |

Values are numbers of non-survivors/survivors (mortality rate, %)

uCysC(−) indicates the absence of tubular injury, and uCysC(+) indicates the presence of tubular injury defined by the indicated cut-off value of the uCysC. Cut-off value (1788 ng/mg uCr): the optimal cut-off value of initial uCysC for predicting AKI. Cut-off value (3389 ng/mg uCr): the optimal cut-off value of initial uCysC for predicting severe AKI. Cut-off value (471.5 ng/mg uCr): the optimal cut-off value of initial uCysC for predicting mortality. Cut-off value (8816 ng/mg uCr): the optimal cut-off value of peak uCysC for predicting AKI. Cut-off value (1736 ng/mg uCr): the optimal cut-off value of peak uCysC for predicting severe AKI. Cut-off value (1260 ng/mg uCr): the optimal cut-off value of peak uCysC for predicting mortality.

^*^P<0.05 vs. uCysC(−)/AKI(−). ^#^P<0.05 vs. uCysC(+)/AKI(−). ^&^P<0.05 vs. uCysC(−)/AKI(+).

**Table S8** Demographic and clinical characteristics and outcomes grouped according to urinary cystatin C and AKI status defined by the optimal cut-off value of the initial uCysC for predicting AKI (n=510)

|  | **uCysC(−)/ AKI(−)** | **uCysC(+)/ AKI(−)** | **uCysC(−)/ AKI(+)** | **uCysC(+)/ AKI(+)** | **P** |
| --- | --- | --- | --- | --- | --- |
| n | 367 (72.0) | 64 (12.5) | 49 (9.6) | 30 (5.9) | N/A |
| Body weight, kg | 4.2 [2.5-10.0] | 2.6 [1.5-7.9]^*^ | 7.0 [2.9-17.3]^#^ | 7.3 [2.0-25.8]^#^ | <0.001 |
| Male, n | 217 (59.1) | 35 (54.7) | 33 (67.3) | 19 (63.3) | 0.558 |
| Illness severity^a^, score | 5 [3-8] | 8 [6-11]^*^ | 7 [5-12]^*^ | 12 [8.8-19]^*#&^ | <0.001 |
| MV, n | 102 (27.8) | 17 (26.6) | 22 (44.9)^*#^ | 21 (70.0)^*#&^ | <0.001 |
| MV Duration, days | 0 [0-0.88] | 0 [0-0.57] | 0 [0-4.25]^*^ | 3.16 [0-5.44]^*#^ | <0.001 |
| Severe AKI^b^, n | 0 (0) | 0 (0) | 17 (34.7)^*#^ | 19 (63.3)^*#&^ | <0.001 |
| ICU LOS, hours | 144.0 [66.0-288.0] | 348.0 [120.0-798.0]^*^ | 156.2 [65.5-312.0]^#^ | 194.6 [112.5-624.6] | <0.001 |
| Death, n | 23 (6.3) | 9 (14.1)^*^ | 5 (10.2) | 11 (36.7)^*#&^ | <0.001 |

Values are median [interquartile range]. Numbers in parentheses denote percentages.

AKI, acute kidney injury; ICU, intensive care unit; LOS, length of stay; MV, mechanical ventilation.

uCysC(−) indicates the absence of tubular injury, and uCysC(+) indicates the presence of tubular injury defined by the optimal cut-off value of the initial uCysC for predicting AKI (1,788 ng/mg uCr).

^a^Illness severity was assessed by the score for neonatal acute physiology in critically ill neonates and the pediatric risk of mortality III score in critically ill children. ^b^Severe AKI was defined as KDIGO stages 2 and 3.

^*^P<0.05 vs. uCysC(−)/AKI(−). ^#^P<0.05 vs. uCysC(+)/AKI(−). ^&^P<0.05 vs. uCysC(−)/AKI(+).
