# Supplementary figures and images for: Subclinical acute kidney injury is associated with adverse outcomes in critically ill neonates and children
Source: Crit Care. 2018 Oct 10;22:256. doi: 10.1186/s13054-018-2193-8 (PMC6180629; doi:10.1186/s13054-018-2193-8)

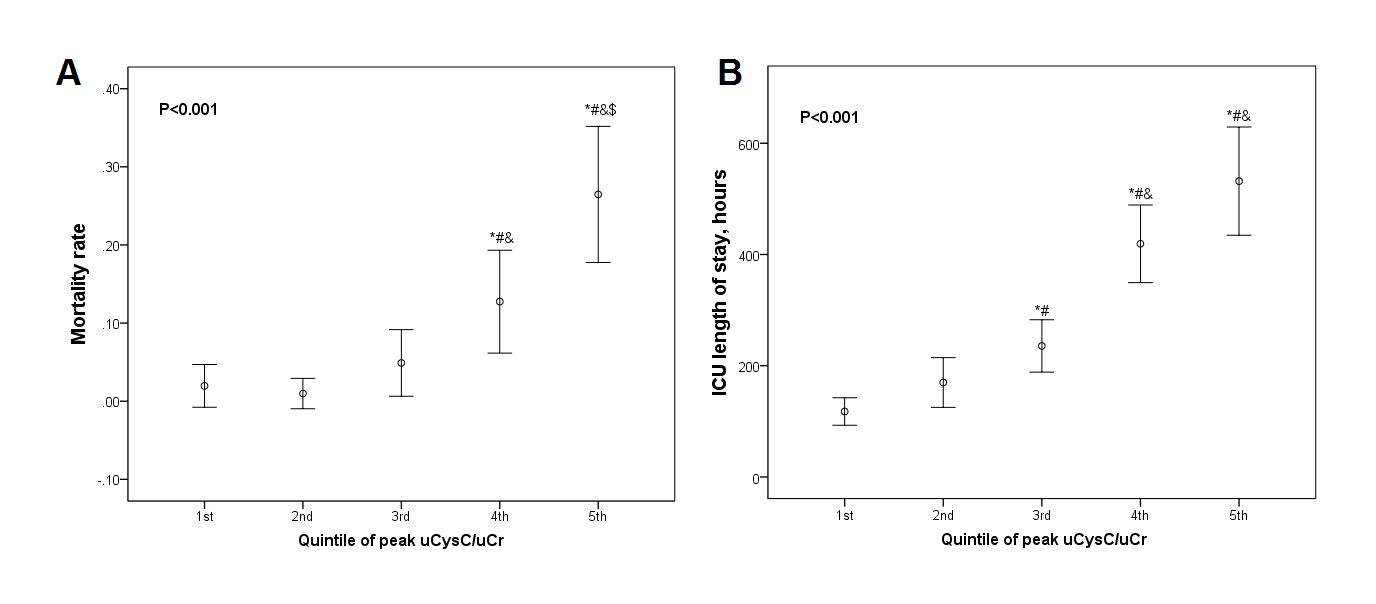

Supplement: Supplementary file 2 — Figure S1. Comparison of ICU mortality (A) and the length of ICU stay (B) by quintile of peak value of urinary cystatin C in critically ill neonates and children (n = 510). The peak values of urinary CysC were divided into quintiles, n = 102 in each group. P value refers to comparison among five groups. *P < 0.05 vs. the first; #P < 0.05 vs. the second; &P < 0.05 vs. the third; $P < 0.05 vs. the fourth quintile. A Error bars represent the mortality rate and 95% confidence interval. B Error bars represent the mean and 95% confidence interval. (TIF 72 kb) [file 13054_2018_2193_MOESM2_ESM.tif]
